# Navigating cardiac arrest together: A survivor and family-led co-design study of family needs and care touchpoints

**DOI:** 10.1016/j.resplu.2024.100793

**Published:** 2024-10-16

**Authors:** Matthew J. Douma, Samina Ali, Tim A.D. Graham, Allison Bone, Sheila D. Early, Calah Myhre, Kim Ruether, Katherine E. Smith, Kristin Flanary, Thilo Kroll, Kate Frazer, Peter G. Brindley

**Affiliations:** a School of Nursing, Midwifery and Health Systems, University College Dublin, Ireland; b Department of Critical Care Medicine, Faculty of Medicine and Dentistry, University of Alberta, Canada; c Department of Pediatrics, Faculty of Medicine and Dentistry, University of Alberta, Canada; d Department of Emergency Medicine, Faculty of Medicine and Dentistry, University of Alberta, Canada; e Barwon Health, University Hospital Geelong, Victoria, Australia; f Australia and New Zealand Intensive Care Research Centre, School of Public Health and Preventive Medicine, Monash University, Australia; g Forensic Nurse Consultant, Surrey, British Columbia, Canada; h Faculty of Medicine and Dentistry, University of Alberta, Canada; i Alberta Health Services, Alberta, Canada; j Patient Partner, Portland, OR, USA

**Keywords:** Cardiac arrest, Qualitative research, Patient engagement, Family-centered care, Touchpoint mapping, Co-design

## Abstract

**Introduction:**

This study aimed to i) identify the care needs of families experiencing cardiac arrest; and ii) co-identify strategies for meeting the identified care needs. Cardiac arrest survivors and family members (of survivors and non-survivors) were engaged as “experience experts,” collaborators and co-researchers in this study.

**Methods:**

A qualitative study using semi-structured interviews of cardiac arrest survivors and family members was conducted. Participants were recruited from the membership of the Family Centred Cardiac Arrest Care Project. Interviews were recorded, transcribed, and analysed using Framework analysis.

**Results:**

Twenty-eight participants described 22 unique cardiac arrest events. We identified five primary care need themes: 1) “Help us help our loved one”; 2) “Work with us as a cohesive team”; 3) “See us: treat us with humanity and dignity”; 4) “Address our family’s ongoing emergency”; and 5) “Help us to heal after the cardiac arrest” as well as 29 subordinate care need themes. We performed touchpoint mapping to identify key moments of interaction between patients and families, and the health system to highlight potential areas for improvement, as well as strategies for meeting family care needs.

**Conclusion:**

Our participants identified varied family care needs during and long after cardiac arrest. Fortunately, many proposed strategies are inexpensive and have low barriers to adoption. However, some unmet care needs identified suggest larger systemic issues such as service gaps that leave families feeling abandoned and isolated. Overall, our findings suggest that care during and after cardiac arrest are critical components of a comprehensive cardiac arrest care system.

## Highlights

• Co-design study exploring family needs during and after cardiac arrest with survivors and family members.
• Patient and public involvement and engagement throughout research process.
• Touchpoint mapping used to visualize family journey in cardiac arrest care.
• Five primary themes of family care needs identified.

## Graphical abstract

**Figure:**
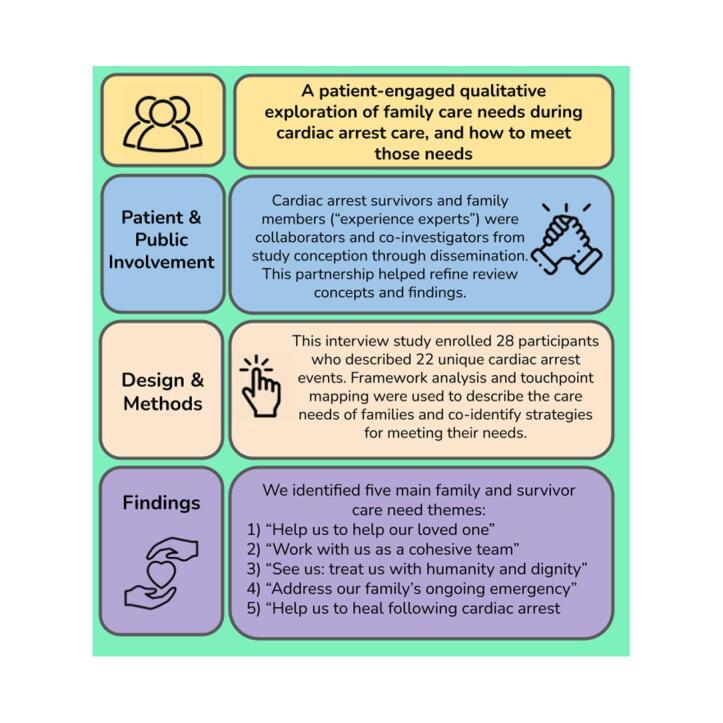

## Introduction

Every year, millions of family members suffer emotional shock and distress when a loved one experiences unexpected cardiac arrest.^1^, ^2^ This can include discovering their family member in extremis, initiating rescue attempts, watching pre-hospital or in-hospital resuscitation, enduring the uncertainty of the post-arrest period, becoming a caregiver, or experiencing the premature death of a loved one.^3^, ^4^, ^5^, ^6^

Recent research has shed light on the profound impact that cardiac arrest has on families. A systematic review highlighted that family members often experience significant psychological distress, including symptoms of post-traumatic stress disorder, anxiety, and depression.^7^ Families of cardiac arrest survivors report feeling 'trapped in a disrupted normality', struggling to adapt to new roles and responsibilities.^8^ Moreover, nearly 50 % of family members of out-of-hospital cardiac arrest patients met criteria for post-traumatic stress disorder, emphasizing the long-term psychological impact.^9^ These studies underscore the need for a comprehensive understanding of family experiences and care needs throughout the cardiac arrest journey.

While families and patients are often grateful for the efforts of healthcare workers, intra-arrest care has also been described as dehumanising and traumatic, punctuated by despair and uncertainty.^10^, ^11^ There can also be substantial and long-lasting emotional and physiological distress, regardless of outcome.^12^, ^13^ Fortunately, resuscitation systems have started to explore the experience of survivors, caregivers, and families.^14^, ^15^, ^16^, ^17^ However, the experiences and needs of families and survivors are insufficiently understood.

This exploratory study was designed to i) identify the care needs of families experiencing cardiac arrest; and ii) co-identify strategies for meeting the identified care needs. We did this by including co-investigators and co-collaborators (“experience experts“) with lived experience of personal and familial cardiac arrest, as members of the research team, not only as participants.

## Methods

### Study design

Our overall project, and this study, were guided by a pragmatist research paradigm; meaning it focuses on practical solutions to real-world problems, using whichever methods are most effective for addressing the research question.^18^ This study employed a qualitative descriptive approach.^19^ This approach is suited to obtaining descriptions of phenomena and is often used when researchers seek to describe an experience or event in easily understood language. It allows for a rich, straightforward description of the experience or event from the perspective of those involved, making it ideal for exploring family experiences of cardiac arrest care.

### Participant recruitment and setting

Potential interview participants were contacted via email invitation from the existing membership of the Family Centred Cardiac Arrest Care Project, a larger program of related research employing co-design and health design thinking methods.^17^ We used purposive sampling to obtain a variety of interviewee demographics and experiences, including the age of the person experiencing cardiac arrest, family member relationship, location (in or out of hospital), and the outcome of resuscitation. This sampling strategy aimed to capture a wide range of perspectives and experiences.^20^

We chose to recruit from the existing Family Centred Cardiac Arrest Care Project membership due to the established rapport and trust with these individuals, which we believed would facilitate more open and in-depth discussions about their experiences. This approach aligns with purposive sampling strategies in qualitative research, where participants are selected based on their ability to provide rich, relevant data.^21^

### Data collection

Interviews were guided using a semi-structured interview tool that was developed from a literature review as well as input and piloting with our experience experts. The interview guide covered topics such as the cardiac arrest experience, interactions with healthcare providers, perceived needs during and after the event, and suggestions for improvement (see online supplement).

Interviews took place between April 2021 and February 2022 using the Zoom™ videoconferencing platform. This method was chosen due to the ongoing COVID-19 pandemic and to allow for the geographic diversity of participants. Interviews were conducted by male author MJD (a 41-year-old male whose grandfather was a cardiac arrest victim, an emergency and critical care nurse with training in qualitative and quantitative research methods, and a PhD candidate at the time of the research). Data collection, analysis, and research integrity were overseen by author PGB (professor and intensive care physician).

Interviews were audio-recorded and transcribed verbatim. Field notes were taken during and immediately after each interview to capture non-verbal cues and initial reflections (see online supplement Fig. 3 for a sample of field notes). Interviews continued until we were able to adequately answer our study aims, as determined by the research team when no additional themes or insights were being identified from the data.^20^

We determined data saturation using the approach outlined in literature.^22^ This involved two key aspects: code saturation and meaning saturation. The research team discussed saturation after every three interviews, reviewing new codes and interpretations. We continued interviewing until both code and meaning saturation were achieved, which occurred after 26 interviews.

### Data analysis

We used framework analysis, as described and developed by methodologists Ritchie and Gale.^23^, ^24^ This method is well-suited for applied policy research and allows for both a priori and emergent theme identification. The process involves five stages: familiarization, identifying a thematic framework, indexing, charting, and mapping and interpretation (see online supplement Table 5 for further description). This approach allowed us to systematically analyze our data while remaining flexible to new insights. It also facilitated comparison across cases (individual interviews) and themes, enabling us to identify patterns in the data while maintaining the connection to each participant’s interview narrative.

Our coding approach combined deductive and inductive methods. We started with a priori codes from the Family Centred Cardiac Arrest Care conceptual framework^16^ but remained open to new codes emerging from the data. Two researchers independently coded each transcript, then met to discuss and resolve any discrepancies, enhancing the reliability of our coding. As we progressed through the indexing stage, we regularly reviewed and refined our coding framework. In the charting stage, we summarized the coded data into a matrix, allowing us to compare themes across cases. Finally, in the mapping and interpretation stage, we looked for patterns, associations, and explanations in the data, moving from descriptive accounts to more interpretive analysis. Throughout this process, we engaged in regular team discussions to challenge and refine our interpretations.

**Fig. 1:**
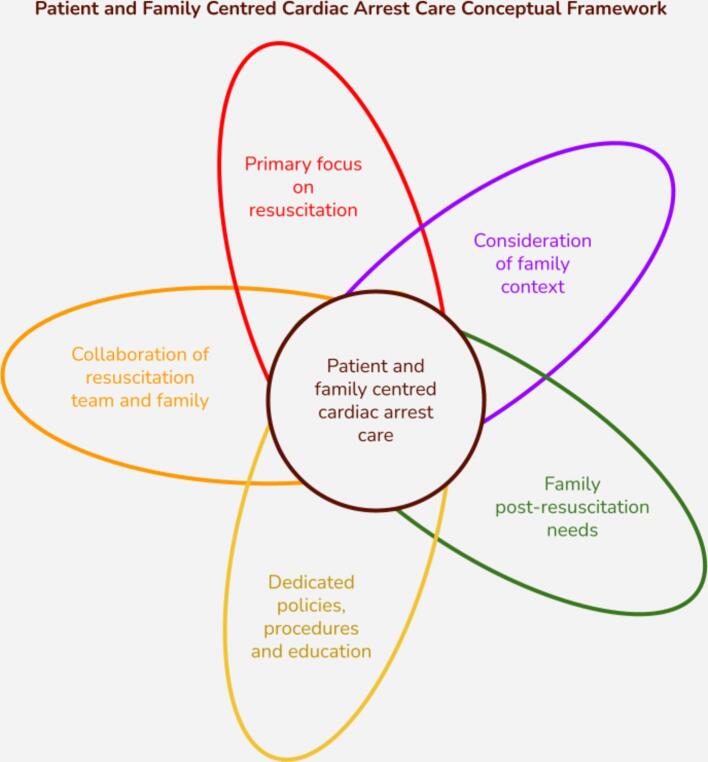
Family Centred Cardiac Arrest Care Framework.

The framework we used consists of five key components: (1) Focus on the family member in cardiac arrest, (2) Collaboration of the resuscitation team and family, (3) Consideration of family context, (4) Family post-resuscitation needs, and (5) Dedicated policies and procedures.^9^ This framework guided our study design, data collection, and initial analysis, providing a structured initial approach. However, we remained open to new themes and insights beyond this framework. A visual representation of the framework is provided in Fig. 1.

Data processing involved the following steps. Audio recordings of interviews were automatically transcribed. The primary investigator (MJD) reviewed all transcripts while listening to the audio recordings to correct any errors and ensure accuracy. Transcripts were then imported into Taguette software for coding.^25^ Personal identifiers were removed from the transcripts to maintain participant confidentiality. The research team, including experience experts, developed a coding framework based on our initial review of transcripts. Two researchers independently coded each transcript, discussing and resolving any discrepancies to ensure consistency. Researchers and experience experts held meetings to discuss and refine the analysis, reach consensus on themes, and finalize the extracted themes.^26^

Furthermore, we employed journey mapping, a user-centered design technique, to visualize the family experience of cardiac arrest care.^27^ This method involves creating a visual representation of the user's journey through a service or experience, highlighting key touchpoints and emotional responses. We adapted this approach to the cardiac arrest context, focusing on family experiences from the initial event through to long-term outcomes. The mapping process involved synthesizing data from our interviews to identify key stages, touchpoints, and emotional experiences reported by families. We then created a visual representation of this journey, highlighting critical moments of interaction between families and the healthcare system. This approach allows for a holistic view of the family experience and can reveal opportunities for service improvement.^28^

### Ethical considerations

This study was approved by the University of Alberta Institutional Review Board (Pro00114528) and conforms with all local and national guidelines for the conduct of patient-oriented research, including the engagement of co-researcher experience experts who were offered honoraria for their involvement.^29^, ^30^ Informed consent was obtained from all participants, and they were assured of confidentiality and their right to withdraw from the study up until the point of transcript anonymization.

### Trustworthiness

To ensure the trustworthiness of our findings, we employed the criteria outlined by Lincoln and Guba^31^: credibility, transferability, dependability, and confirmability. Credibility was enhanced through prolonged engagement with the data, member checking with participants, and investigator triangulation. Transferability was addressed through a thick description of the research context and participant characteristics. Dependability was ensured through an audit trail of our research process and decisions. Confirmability was supported by researcher reflexivity and the involvement of multiple researchers in data analysis. This manuscript is reported in accordance with best practices in patient and public involved research and qualitative research reporting. See online supplement GRIPP2 and SRQR.

## Results

Twenty-eight participant interviews were conducted to achieve adequate diversity of representation. Of 38 eligible participants emailed, 8 did not respond and 2 declined, stating a lack of time or availability for not participating. Following enrollment, 28 participants attended 26 semi-structured interviews representing 22 unique cardiac arrest experiences. Interview duration ranged from 34 to 126 min. See Table 1 for participant demographics.

**Table 1:** Participant demographics.

Participant description	N (%)
Survivor	3
Family member of survivor	14
Family member of non-survivor	11
Total participants	28

Cardiac arrest setting	
Initial cardiac arrest out-of-hospital	12
Initial cardiac arrest in-hospital	6
Subsequent re-arrest in-hospital	8
Family in-hospital arrest experience	14

Cardiac arrest aetiology	
Confirmed medical	20
Unconfirmed medical	2*

Person who experienced cardiac arrest’s age	
Older adult (>65 years)	10
Adult (18 to 64 years)	10
Child (<18 years)	2

Patient outcome	
Died out-of-hospital	6
Died in-hospital	5
Alive and living independently	7
Alive and living dependent on caregivers	4

Family members	
Partner	12
Adult child	8
Parent	2
Sibling	2
Close friend	1
Years since cardiac arrest (mean, range)	6 (1 to 17)

Country of residence of participant	
Canada	13
United States of America	7
Australia	4
United Kingdom	4

Our interview participants all had direct experience with cardiac arrest: 3 were survivors, 14 were family members of survivors, and 11 were family members of non-survivors. Sixteen of the cardiac arrests (initially) occurred out of hospital and six within hospital. Twenty of the cardiac arrests were of medical aetiology while two were described as having unknown cause and were associated with minor traumatic injury (falls from standing with presumed medical etiology).

This qualitative study identified five primary care need themes from the experiences of families who have encountered cardiac arrest in a loved one, and 29 subordinate themes. A summary of primary and subordinate themes are described below in Table 2. A selection of exemplary quotes can be found in Table 3. See Table 6 in the online supplement for a complete table of our findings.

1. “Help us help our loved one”

**Table 2:** Findings: Primary and Subordinate Themes.

Primary Theme	Subordinate Theme
1. Help us help our loved one	(a) Recognize the cardiac arrest (b) Initiate first response (c) Support professional rescuers (d) Focus on our loved-one, at first

2. Work with us, as a cohesive team	(a) Support families in their presence or absence (b) Allow shared decision making (c) Acknowledge family's knowledge and insights (d) Prepare families for what to expect (e) Be culturally competent (f) Recognize that survival is not the goal for everyone

3. See us, treat us with humanity and dignity	(a) Prepare families for what they might see (b) Acknowledge family experiences (c) Avoid seemingly arbitrary and senseless restrictions; they feel cruel and hurtful (d) Remember to always attempt to contact and update families (e) Provide basic accommodations for families (f) Let families be present as much as possible (g) Acknowledge my loved one's humanity through words (h) Help us fulfil basic needs while we attend to our loved one (i) Give us space to be a family together

4. Address our family's ongoing emergency	(a) Recognize that while the patient's emergency resolves, the family's emergency builds (b) Help us care for kids and dependents (c) Defuse our acute grief and distress with us

5. Help us to heal, after the cardiac arrest	(a) Navigate the after-arrest world (b) Train us to be caregivers (c) Give us resources for healing (d) Find our way in a disjointed healthcare system (e) Partner with us in expressions of gratitude (f) Be in the service of others (g) Find peer support and community

**Table 3:** Select findings: Care needs of families experiencing the cardiac arrest of a loved one.

**Primary care need themes**	**Subordinate care need themes**	**Exemplary quotes**
1) Help us help our loved one	1a) Recognize the cardiac arrest	*“He just kept making the sound and it got a little more almost urgent sounding, you know, it was just a bizarre sound. Then it was more like, it seemed like he wasn’t able to breathe very well. So, then I listened, put my head to his chest and, you know, tried to tell whether he was breathing or, you know, just if I could hear anything, and I couldn't hear a heartbeat. And this was really early in the COVID pandemic. And I didn't know what agonal breathing was, or what any of the signs are really, for cardiac arrest. So I didn't know what was happening. But because it was the time that it was, and it sounded respiratory, I assumed, you know, he, maybe he's got COVID, and he's having trouble breathing.”* Partner of survivor Participant #11
	1d) Focus on our loved-one, at first	*“Yeah, like, help, needs to arrive and help needs to arrive fast. And like in that moment, I think I wasn't really concerned about myself, or what I needed. I just wanted him to be ok.”* Partner of non-survivor Participant #5

2) Work with us, as a cohesive team	2a) Support families in their presence or absence	*“I was there the whole time. I got to see the whole resuscitation and I watched the team work so hard. I knew they cared for him and I know he did not suffer. And there’s no way they could have made me leave. I am so glad I could be there. I spoke to him and held his hand even. I was there the whole time and the nurse stayed with me too, the whole time. I was caring for him and she was caring for us.”* Son of non-survivor Participant #9
	2f) Recognize that survival is not the goal for everyone	*“I knew there was nothing more they could do. I knew, I knew at that point it had been too long and she wouldn’t have wanted anymore. They were still going, trying as hard as they could, but I knew it was time to stop, like, like, there’s no chance, it had been almost an hour. I told them I thought it was enough. I wanted to say good bye then. The doctor in charge talked to me then and kind of confirmed with me and spoke to the rest of the people there and they stopped the CPR and turned off the machines and we got to be there and say goodbye like you should.”* Son of non-survivor Participant #24

3) See us, treat us with humanity and dignity	3c) Avoid seemingly arbitrary and senseless restrictions; they feel cruel and hurtful	*“They set a timer for us. One at a time, for fifteen minutes. They would not let us grieve together or be a family, they singled us out. And there was lots of room and no one was doing anything with, like she was stable then and comatose. There was no reason to treat us like that. It was terrible.”* Daughter of survivor Participant #14
	3d) Remember to always attempt to contact and update families	*“Please call me to tell me this has happened. Like, please make some effort to let families know. I was at home, just doing whatever, not important and he is living his last moments and I don’t get to be a part of them because no one thinks to even call me. I have so much guilt because I was going about my life while my [partner] was having their last moments, alone, probably in pain, probably terrified and I was probably folding laundry in front of the television.”* Partner of non-survivor Participant #3
	3g) Acknowledge my loved one’s humanity through words	*“And the ICU nurse, his name was X. He would talk with him about those pictures. And he was doing it in his nursing capacity. What he was doing was trying to find out what his cognitive ability and all of that was, right? But he did it in such a humane, compassionate way, as well, where he would just talk to him and just say, “Oh, are these your kids? What are their names? How old are they? And what school do they go to? Just things that are really designed to jog his memory and evaluate where his memories are, but also just talk to the patient about their life. And, you know, he was the only person I think that did that. And you know, without the family able to be there with him, that was it you know? And that has stuck with me as a really shining example of, of what to do and how to do it in those situations.”* Partner of survivor Participant #11
	3h) Help us fulfil basic needs while we attend to our loved one	*“You know, like there, there weren't any chairs, and the lights weren't even on. It's just sort of like, it's almost like when you're in a classroom after school hours, and everything is put away. Yeah. It doesn't feel like a space that you're supposed to be in. It doesn’t feel like it is a space that’s supposed to be used.”* Son of survivor Participant #13
	3i) Give us space to be a family together	*“I think the family room was actually nice. We charge our phones, we could have a cup of tea and be together. Having a place where we felt comfortable when we weren’t in the actual room, was really nice. And for the first night, they got me a recliner chair and a pillow and flannel and that was really great.”* Daughter of survivor Participant #16

4) Address our family’s ongoing emergency	4b) Help us care for kids and dependents	*“I’ve got kids and my mother to take care of. And now my husband, he barely survived a cardiac arrest. Right? I needed a lot of help and I had none. Our house was a huge mess from the first responders, they had to break the door down. My kids were at school, my mother was at home alone, who knows what she understood. They all depend on me and I cannot be everywhere and be everything to everyone.”* Partner of survivor Participant #19
	4c) Defuse our acute grief and distress with us	*“So after they called the code they brought us into the room. All the doctors and nurses were walking out except for one. She said we could have twenty minutes to say goodbye and then we had to leave. I couldn’t believe it, I was so distraught, like we just had a total whirlwind. This was the worst day of our lives. I went from asleep to trying to resuscitate [female partner], to them dying in the hospital and we were being totally abandoned. We had no idea why she died, I couldn’t make sense of anything, I didn’t know what to do or what to think. I was wondering where they were going, because they’re not done, done. We still needed their help, but I don’t think they see it that way.”* Partner of non-survivor Participant #22

5) Help us to heal, after the cardiac arrest	5a) Navigate the after-arrest world	*“No, it has not ended. I think that it is a process that will never be done because, you know, we live with the consequences of our actions prior and post and so it's something that you live with the regret of the night before he left, you live with the regret of not going directly to the school and perhaps, you know, helping them apply the AED while he was still on the gym floor. And then afterwards it's years of dysfunction and broken family events because of missing him and, you know, grieving differently and fighting and divorce and so I think that it goes on way beyond what anybody realises and it impacts people's lives forever.”* Mother of non-survivor Participant #18
	5d) Find our way in a disjointed healthcare system	*“We needed cardiology follow-up and neurological rehabilitation and job retraining. And we needed genetic testing and counselling. And we don’t even have a family doctor. So, we are at home and we don’t even know where to start. It is like, you’re discharged and then you are on your own to figure out what to do and I don’t even know what’s needed and what’s available.”* Partner of survivor Participant #15
	5f) Be in the service of others	*“One of my sister’s friends, her husband had a cardiac arrest in the middle of the night at age 50, and survived. They live in [another province]. And she connected us because this woman was completely lost. We don't talk often, but every time we talk, I always feel better. Because I mean, there's some revisiting trauma, of course, but at the end of it, after it's processed, I feel empowered that I helped someone, and her response is always, nobody else makes me feel better, like you do.”* Partner of survivor Participant #12
	5g) Find peer support and community	*“Peer support is the best, nothing could be more valuable. But, I do wonder if peer follow-up calls need to come from someone who's been there, and quite matched. So if your 90 year old has died you don't call someone whose 30 year old husband died, because it's just different, it just is. It's like a two year old* versus *an eighty year old dying. It's not the same thing. And for very different reasons. It's not the same thing. And you don't compare traumas; one is not worse than the other. It's just about relatability. But for myself, there's some empowerment and healing in being able to be there for someone else to say, this terrible thing happened to us. And I've turned it into a tool for good in some way. Right?”* Partner of survivor Participant #12

This theme encompasses the urgent desire of family members to contribute to their loved one's care during the critical moments of cardiac arrest. It includes recognizing the signs of cardiac arrest, initiating first response, and supporting professional rescuers. One participant described the challenge of recognizing cardiac arrest:

*“He just kept making the sound and it got a little more almost urgent sounding, you know, it was just a bizarre sound. Then It was more like, it seemed like he wasn't able to breathe very well. So, then I listened, put my head to his chest and, you know, tried to tell whether he was breathing or, you know, just if I could hear anything, and I couldn't hear a heartbeat.” (Participant #11, Partner of survivor).*

Another participant emphasized the importance of immediate action:

*“Yeah, like, help, needs to arrive and help needs to arrive fast. And like in that moment, I think I wasn't really concerned about myself, or what I needed. I just wanted him to be ok.” (Participant #5, Partner of non-survivor).*

2. “Work with us as a cohesive team”

This theme highlights families' desire for effective communication and shared decision-making with the healthcare team. It emphasizes the importance of including families in the care process and recognizing their knowledge and insights. One participant expressed the value of being present during resuscitation:

*“I was there the whole time. I got to see the whole resuscitation and I watched the team work so hard. I knew they cared for him and I know he did not suffer. And there's no way they could have made me leave. I am so glad I could be there. I spoke to him and held his hand even. I was there the whole time and the nurse stayed with me too, the whole time. I was caring for him and she [the nurse] was caring for us.” (Participant #9, Son of non-survivor)*

Another participant highlighted the importance of cultural competence:

*“You know it is part of our culture, it is part of our faith that we perform our last rites. We needed some time and some space to perform them. The hospital staff were amazing, they made sure we could perform our last rites and Dad could then pass in peace.” (Participant #17, Daughter of non-survivor).*

3. “See us: treat us with humanity and dignity”

This theme illustrates the need for healthcare providers to respect and cater to the needs of families during their interactions with the medical system. It includes preparing families for what they might see, acknowledging their experiences, and providing basic accommodations. One participant described the shock of seeing their loved one in the hospital and the experience of being unsupported and uninformed:

*“Yeah. But when I came to see him, it was like, he was unconscious, he was comatose, and he just had all these wires sticking out of him. And I remember being nervous to even touch them. Like it was, it was pretty freaky having him presented to me like that. I didn't have any idea he was going to look the way it did.” (Participant #13, Son of survivor)*

Another participant emphasized the importance of acknowledging families:

*“I don't think any of them (health care workers) even talked to me about it. Nobody even said, ”How are you?“ or ”How can we take care of you?“ It also felt really strange like, it felt like there should have been something there but wasn't. It is the single worst experience of my life, I am forever changed and traumatised, and I'm in this hospital which is supposed to be a place of healing and there's nothing for me? No help for me?” (Participant #13, Son of survivor)*

4. “Address our family's ongoing emergency”

This theme articulates a critical need for support mechanisms that extend beyond immediate medical interventions for patients. It recognizes that families experience their own crisis that requires attention and support. One participant explained:

*“At first we needed help for [partner]. We needed the first responders and we needed all the hospital staff. We needed them to fix his heart. But we also needed help. After the initial cardiac arrest, our family was actually in crisis and as he got better, we actually got worse. And I don't want to seem ungrateful because we are so fortunate, but after they saved [male partner], we needed someone to help save us.” (Participant #21, Partner of survivor).*

Another participant highlighted the challenge of managing multiple responsibilities:

*“I've got kids and my mother to take care of. And now my husband, he barely survived a cardiac arrest. Right? I needed a lot of help and I had none. Our house was a huge mess from the first responders, they had to break the door down. My kids were at school, my mother was at home alone, who knows what she understood. They all depend on me and I cannot be everywhere and be everything to everyone.” (Participant #19, Partner of survivor).*

5. “Help us to heal after the cardiac arrest”

This theme revolves around the intricate process of navigating life after such a traumatic event. It includes learning to grieve, be a caregiver, finding peer support, and expressing gratitude. A mother of a non-survivor shared:

*“No, it has not ended. I think that it is a process that will never be done because, you know, we live with the consequences of our actions prior and post and so it's something that you live with the regret of the night before he left, you live with the regret of not going directly to the school and perhaps, you know, helping them apply the AED while he was still on the gym floor.” (Participant #18, Mother of non-survivor).*

Another participant emphasized the importance of peer support:

*“Peer support is the best, nothing could be more valuable. But, I do wonder if peer follow-up calls need to come from someone who's been there, and quite matched. So if your 90 year old has died you don't call someone whose 30 year old husband died, because it's just different, it just is. It's like a two year old versus an eighty year old dying. It's not the same thing. And for very different reasons. It's not the same thing. And you don't compare traumas; one is not worse than the other. It's just about relatability.“ (Participant #12, Partner of survivor).*

Our qualitative findings directly informed the development of the journey map. Each theme represents a critical aspect of the family experience, which we integrated into different stages of the journey. For example, the theme 'Help us help our loved one' is prominently featured in the early stages of the journey map, reflecting the immediate needs of families during the cardiac arrest event. The theme 'Address our family's ongoing emergency' is represented in the middle stages of the journey, highlighting the evolving needs of families as the initial crisis passes. The journey map thus provides a visual synthesis of our thematic analysis, illustrating how family needs change and persist throughout the cardiac arrest care trajectory.

### Touchpoint mapping

**Fig. 2:**
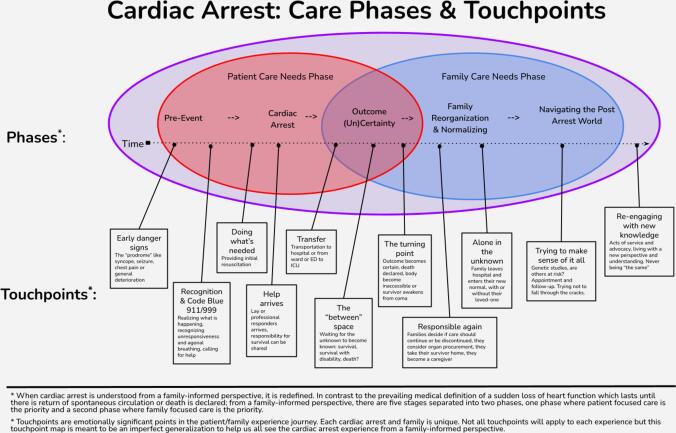
Touchpoint Map.

Our touchpoint mapping and analysis illustrated the cardiac arrest event consisting of a patient care needs phase which overlaps with a family care needs phase. Within the patient care phase, there were three subphases: a pre-event subphase, the cardiac arrest subphase and the outcome uncertainty subphase. Within the family care phase there were also three subphases: an outcome uncertainty subphase, a family reorganization subphase and a navigating the post-arrest world subphase (see Fig. 2).

The touchpoint mapping outlines a patient and family's journey through cardiac arrest, commencing with the “prodrome” (early signs such as syncope, seizures, chest pain, or general deterioration), through the immediate resuscitative efforts, the liminal “between” space filled with uncertainty, the turning point of outcome certainty, and finally the period of adjustment as the family departs from the hospital, transitioning into their new normal. See Table 4, for a co-designed table of strategies to for meeting the care needs of families and table 7 in the online supplement for an expanded table of strategies.

**Table 4:** Summary of co-designed strategies for meeting the care needs of families experiencing cardiac arrest.

Primary care need theme	Strategies Summary
Help us help our loved one	Implement comprehensive public education on cardiac arrest recognition and bystander CPR Enhance dispatcher-assisted CPR protocols and create supportive technologies for family first responders Integrate cardiac arrest preparedness into routine healthcare and high-risk patient management

Work with us, as a cohesive team	Develop and implement family-centred resuscitation protocols with dedicated family liaison roles Create clear guidelines on family presence during resuscitation and shared decision-making models Establish regular collaborative forums for healthcare providers, survivors, and families to improve care processes

See us, treat us with humanity and dignity	Implement family-centred care approaches, including rounds and needs assessments Create supportive physical and emotional environments for families during the crisis Develop comprehensive staff training on trauma-informed care and compassionate communication

Address our family’s ongoing emergency	Establish a multi-faceted support system including hotlines, care coordination, and community partnerships Implement proactive outreach and education programs for families on managing the crisis Develop resources to address practical needs (e.g., childcare, financial counselling) during the emergency period

Help us to heal, after the cardiac arrest	Establish long-term follow-up programs addressing physical, psychological, and social needs of survivors and families Create diverse support and education initiatives for different family roles and outcomes Develop research and community-based programs to understand and address long-term impacts on families

## Discussion

Through in-depth interviews and thematic analysis with family members with lived experience of cardiac arrest, we have described their experiences and care needs, mapped them and generated a co-designed inventory of potential strategies to address them. Our findings align with and expand upon previous research in this area.

The theme “Help us help our loved one” echoes findings described by Bremer et al.^32^, who emphasized the intense desire of family members to contribute during cardiac arrest. Our study further emphasizes the importance of empowering families through tele-CPR, first aid training, support in recognizing cardiac arrest, and performing initial CPR. In the out of and in-hospital settings, both recognition and timely rescue is a primary need.

“Work with us as a cohesive team” resonates with the concept of family-centered care, increasingly recognized in critical care settings.^33^ Our findings underscore the need for healthcare providers to view families not just as bystanders, but as integral members of the care team. This aligns with recent guidelines encouraging family presence during resuscitation,^34^ but our study suggests that more comprehensive integration of families into the care process is needed, supporting presence, absence and aftercare.

The theme “See us: treat us with humanity and dignity” highlights the profound impact that interactions and images can have on families' experiences. This aligns with research by Burns et al.^35^, who described how the appearance of a cardiac arrest survivor can be distressing for their family and emphasizes the significance of healthcare workers' communication. Our study provides specific examples of how healthcare providers can acknowledge and support families, even in high-stress situations.

“Address our family's ongoing emergency” expands on previous research by highlighting that the crisis for families builds during and after the resuscitation phase. This aligns with studies where families felt isolated and ignored during and after resuscitation.^19^, ^36^ They experience a disrupted normality^8^ and an abrupt disappearance of the healthcare system's support.^37^

The final theme, “Help us to heal after the cardiac arrest,” underscores the long-lasting impact of cardiac arrest on families, regardless of the patient's outcome. This echoes findings from Naber et al.^38^, who report reduced neuropsychological functioning in survivors along with high rates of anxiety, depression, and post-traumatic stress. Families of non-survivors experience an increased incidence of major depressive episodes, panic disorder, post-traumatic stress disorder, and other psychiatric conditions.^39^ Our study adds to this by offering specific suggestions on how healthcare systems can support families in their long-term healing process.

Our touchpoint mapping approach provides a novel visualization of the cardiac arrest journey from the family's perspective. This builds on previous work using journey mapping in healthcare^40^ but applies it specifically to the cardiac arrest context. This approach could be valuable for healthcare providers and system designers in identifying key points for intervention and support.

### Limitations and methodological considerations

This study has several limitations. First, our sampling method and sample was relatively homogeneous in terms of socioeconomic status and cultural background, which may limit the transferability of our findings to more diverse populations. Second, as a qualitative study, our findings represent the experiences and perspectives of a relatively small number of participants and may not be generalizable to all families experiencing cardiac arrest. Third, the use of video conferencing for interviews, while necessary due to the COVID-19 pandemic, may have influenced the depth of rapport built with participants and potentially affected the richness of data collected. Finally, despite efforts to minimize bias through collaborative analysis and researcher review, the experience experts perspectives may have influenced our findings.

## Conclusion

Our participants identified varied family care needs during and long after the cardiac arrest of a family member. The touchpoint map identifies key experiences that can be the focus of improving cardiac arrest care. Care improvements may be inexpensive and simple to address, such as providing written information, a place to rest, speak in private or charge a phone, and telephone or virtual follow-up. Some of the unmet care needs suggest larger systemic issues, such as family debriefing after resuscitation to reduce experiences of abandonment and isolation and the inclusion of post-arrest follow-up to promote family wellness. Our findings suggest that the integration of care for families is an essential component of cardiac arrest care.

Future research should focus on implementing and evaluating the strategies suggested by our participants. There is also a need for larger, more diverse studies to confirm and expand upon our findings. Ultimately, addressing the needs of families should be recognized as a core component of high-quality cardiac arrest care.

## Preprint

This project exists on a preprint server, however, this study does not exist in preprint available to the public.

## Artificial intelligence

AI was not used in the execution of this research or preparation of this manuscript.

## CRediT authorship contribution statement

**Matthew J. Douma:** Conceptualization, Methodology, Investigation, Formal analysis, Writing – original draft, Project administration. **Samina Ali:** Methodology, Validation, Formal analysis, Writing – review & editing. **Tim A.D. Graham:** Validation, Formal analysis, Writing – review & editing. **Allison Bone:** Validation, Formal analysis, Writing – review & editing. **Sheila D. Early:** Validation, Formal analysis, Writing – review & editing. **Calah Myhre:** Validation, Formal analysis. **Kim Ruether:** Validation, Formal analysis. **Katherine E. Smith:** Validation, Formal analysis. **Kristin Flanary:** Validation, Formal analysis. **Thilo Kroll:** Supervision, Writing – review & editing. **Kate Frazer:** Supervision, Writing – review & editing. **Peter G. Brindley:** Supervision, Methodology, Validation, Formal analysis, Writing – review & editing.

## Declaration of competing interest

The authors declare that they have no known competing financial interests or personal relationships that could have appeared to influence the work reported in this paper.
