# Supplementary material for: Navigating cardiac arrest together: A survivor and family-led co-design study of family needs and care touchpoints
Source: Resusc Plus. 2024 Oct 16;20:100793. doi: 10.1016/j.resplu.2024.100793 (PMC11530864; doi:10.1016/j.resplu.2024.100793)
Supplement: Supplementary Data 1 [file mmc1.docx]

### Online supplement

### A patient-engaged qualitative exploration of family care needs during cardiac arrest care, and how to meet those needs

Table 5. Description of the Framework Analysis

| Stage | Description | Team Members |
| --- | --- | --- |
| 1. Familiarisation with the data | Researchers immersed themselves in the data by reading transcripts and listening to audio recordings. | MJD & PGB |
| 2. Constructing an initial thematic framework | Based on the FCCAC conceptual framework and initial readings, a preliminary coding structure was developed. | MJD & PGB |
| 3. Indexing and sorting the data | Transcripts were coded using the initial framework, with new codes were added as they emerged. | MJD, CM & KES |
| 4. Charting | Data were summarized and arranged in a matrix of themes and subtheme. Themes were also renamed by experience experts. | MJD, SA, KF, TAG, AB, SDE, CM, & KR |
| 5. Mapping and interpreting the data | Researchers looked for patterns, associations, and explanations in the data. | MJD, SA, TAG, AB, KF, SDE, KR, CM, KES & PGB |
| FCCAC: Family centred cardiac arrest care | | |

**Figure 3. Field Notes Examples – Interview 3 (Family Member of Survivor)**

**Date:** Feb 2

**Interview Duration:** 45 minutes
**Location:** Participant's home
**Participant Code:** FM05

**Non-verbal Cues:**

- The participant seemed hesitant at the beginning, crossing their arms and looking down frequently.
- During the discussion about the immediate moments following the cardiac arrest, the participant’s hands began to fidget, and their voice quivered slightly.
- As the interview progressed, particularly when recalling the recovery process, the participant relaxed, leaning back in their chair and maintaining more eye contact.

**Initial Reflections:**

- Emotional weight seemed palpable when discussing the uncertain period immediately following the arrest. The fidgeting may indicate unresolved tension or trauma.
- The participant’s shift in body language suggests a feeling of relief or acceptance when moving from discussing the event to recovery.
- The strong emotional response highlights the enduring impact cardiac arrest has not only on survivors but on family members, even during recovery.

**Field Notes – Interview 14 (Non-survivor Family Member)**

**Date:** Feb 17

**Interview Duration:** 65 minutes
**Location:** University office (workplace) private
**Participant Code:** NSFM07

**Non-verbal Cues:**

- The participant entered the interview room with a noticeable heavy sigh, immediately sitting in a slouched position.
- They avoided eye contact for much of the discussion, especially when speaking about the hospital staff's response during the event.
- When discussing the time after the arrest, the participant’s voice became quieter, and they paused frequently to regain composure.

**Initial Reflections:**

- There was a sense of unresolved frustration or sadness when talking about the medical response to the arrest.
- Avoidance of eye contact might indicate discomfort with revisiting the experience, or it could be a response to feelings of helplessness.
- Pauses and quieter speech reflect deep emotional pain, and this might be a point for further thematic analysis on how families cope with non-survivor outcomes.

**Field Notes – Interview 22 (Cardiac Arrest Survivor)**

**Date:** April 2

**Interview Duration:** 85 minutes
**Location:** Home, kitchen loud with kids in background
**Participant Code:** S03

**Non-verbal Cues:**

- The participant smiled frequently, especially when recalling moments of survival and recovery.
- They spoke animatedly with their hands when discussing the efforts of the paramedics, indicating a strong sense of gratitude.
- At the mention of the family’s reaction, they briefly looked down and lowered their voice, as if reflecting on the emotional toll it took on their loved ones.

**Initial Reflections:**

- The participant’s upbeat demeanor and hand movements suggest a sense of empowerment or renewed appreciation for life post-arrest.
- The emotional shift when discussing family members points to an awareness of the secondary trauma experienced by loved ones.
- Survivors might provide valuable insights into how cardiac arrest impacts not only their physical recovery but also relational dynamics with those around them.

Table 6. Findings: Care needs of families experiencing the cardiac arrest of a loved one

| **Primary care need themes** | **Subordinate care need themes** | **Exemplary quotes** |
| --- | --- | --- |
| 1) Help us help our loved one | 1a) Recognize the cardiac arrest | *“He just kept making the sound and it got a little more almost urgent sounding, you know, it was just a bizarre sound. Then it was more like, it seemed like he wasn’t able to breathe very well. So, then I listened, put my head to his chest and, you know, tried to tell whether he was breathing or, you know, just if I could hear anything, and I couldn't hear a heartbeat. And this was really early in the COVID pandemic. And I didn't know what agonal breathing was, or what any of the signs are really, for cardiac arrest. So I didn't know what was happening. But because it was the time that it was, and it sounded respiratory, I assumed, you know, he, maybe he's got COVID, and he's having trouble breathing.”*  Partner of survivor  Participant #11 |
|  |  |  |
|  | 1b) Support lay-resuscitation | *“So she [the emergency call taker] was the one that really knew how to perform CPR. And she told me to do that. So I did, I had to do it on the bed, because he's a lot taller and a lot heavier than me. And I couldn't move him without maybe hitting his head on, you know, on the nightstand or something. So we had to just go from there. And I did CPR for 10 minutes before the emergency services arrived.”*  Partner of survivor  Participant #11 |
|  |  |  |
|  | 1c) Come quickly and relieve us of sole responsibility | *“Time was going so slowly. We had activated the medical emergency team. His nurses were there, but they did not know what to do. He was not breathing well, like he was suffocating and it was like we were all suffocating, while we waited. I prayed for them to come, but they could not come fast enough.”*  Partner of non-survivor  Participant #1 |
|  |  |  |
|  | 1d) Focus on our loved-one, at first | *“Yeah, like, help, needs to arrive and help needs to arrive fast. And like in that moment, I think I wasn't really concerned about myself, or what I needed. I just wanted him to be ok.”*    Partner of non-survivor  Participant #5 |
|  |  |  |
| 2) Work with us, as a cohesive team | 2a) Support families in their presence or absence | *“I was there the whole time. I got to see the whole resuscitation and I watched the team work so hard. I knew they cared for him and I know he did not suffer. And there’s no way they could have made me leave. I am so glad I could be there. I spoke to him and held his hand even. I was there the whole time and the nurse stayed with me too, the whole time. I was caring for him and she was caring for us.”*  Son of non-survivor  Participant #9 |
|  |  |  |
|  | 2b) Provide culturally competent care | *“You know it is part of our culture, it is part of our faith that we perform our last rites. We needed some time and some space to perform them. The hospital staff were amazing, they made sure we could perform our last rites and Dad could then pass in peace.”*  Daughter of non-survivor  Participant #17 |
|  |  |  |
|  | 2c) Consistency in approach, prevent conflict | *“I think we need something like the Patient’s Bill of Rights, but a Family’s Bill of Rights. It would be nice to have, to know what I can ask for as a family member and what can I do? Like what we can expect of each other.”*  Partner of survivor  Participant #11 |
|  |  |  |
|  | 2d) Adapt to unique family needs | *“And then we had, we had one situation where we've sort of been allowed, I think some of the nurses had said, Okay, look, it's okay, you're pretty quiet, you know, you're not having a riot, you can sit by the bed. And then we had a student nurse, clearly just following the rules. As she came up to my dad, and she said, there can only be two of you around the bed. And I think my dad at that point was stressed, upset, worried, all of those things. And he kinda let rip at her, in a quiet, polite way and then there was an issue.”*  Sister of survivor  Participant #10 |
|  |  |  |
|  | 2e) Provide effective communication | *“I have vague recollections of pamphlets being thrust at me at various points…and I'm sure I read them, but I don't think anything stuck in my brain. But the pamphlets didn't stick because I had no brain space to process. For me. It's possible that it makes sense for some, but in the moment, like I remember when we were discharged, I had like all these pieces of paper, and I was like, I don't even know what just put it in the bag. People handed me things at various times. You kinda need both, human connection, real face-to-face and verbal in addition to the paper.”*  Partner of survivor  Participant #12 |
|  |  |  |
|  | 2f) Recognize that survival is not the goal for everyone | *“I knew there was nothing more they could do. I knew, I knew at that point it had been too long and she wouldn’t have wanted anymore. They were still going, trying as hard as they could, but I knew it was time to stop, like, like, there’s no chance, it had been almost an hour. I told them I thought it was enough. I wanted to say good bye then. The doctor in charge talked to me then and kind of confirmed with me and spoke to the rest of the people there and they stopped the CPR and turned off the machines and we got to be there and say goodbye like you should.”*  Son of non-survivor  Participant #24 |
|  |  |  |
|  | 2g) Ask the experts, they know important things about their loved one | *“And then at that time, they asked if [friend] had ever expressed an interest, or ever expressed any feeling about being an organ donor? And and, you know, the whole room just said, well, yes, of course, she would want to be an organ donor. Actually, [friend] in her career in Melbourne, had actually looked after one of the first people to have a liver transplant in Melbourne. And, and so we said, yes, no, absolutely. And he had said that, you know, in light of the significance of her brain injury she may be an excellent donor.”*  Friend of non-survivor  Participant #4 |
|  |  |  |
|  | 2h) Assign us a person; there are many people, but no one for us | *“I think, in many ways, caring for us is caring for her and vica versa, right? Like we want the same things, we want her to survive or have a good death. And I want to be present and involved in the resuscitation if it doesn’t negatively impact the likelihood of survival. You know? You like, assign us a person like a chaplain or a nurse or a social worker, someone who knows what’s going on and can care for us, and you bring us into the resuscitation. And we can answer questions about her medical history and her medications and we can comfort her while the team does the resuscitating. ‘Cause, Jesus Christ, the alternative is you keep me from my last moments with her and you put me in a family room for your convenience and you forget about me. No way, treating families like that is completely unacceptable.”*  Mother of non-survivor  Participant #23 |
|  |  |  |
| 3) See us, treat us with humanity and dignity | 3a) Prepare us for what we will see | *“Yeah. But when I came to see him, it was like, he was unconscious, he was comatose, and he just had all these wires sticking out of him. And I remember being nervous to even touch them. Like it was, it was pretty freaky having him presented to me like that. I didn't have any idea he was going to look the way it did. I had no idea what any of the machinery was even doing to him. I felt I wasn't prepared for it. It was terrifying.”*    Son of survivor  Participant #13 |
|  |  |  |
|  | 3b) Know that separating and excluding us feel likes mistreatment | *“So they're like “What's your name? What's your relationship to the patient?” and then of course they take you to the family room. And we waited in the family room, which incidentally was no nicer than a cell. It was literally hard chairs and a room with four walls, no natural light. And we waited for about two hours before anyone came to say anything and then the consultant came out and he was lovely but he was quite bleak. Then we waited another three hours, to the point where I then got up and went to reception and said, “my sister, you know, what is happening?” and they were like, “Oh, she went at least two and a half hours ago, up to cardiac intensive care.” And we've been sitting there, absolutely biting our nails down to the quick, you know and they totally forgot about us.”*  Sister of survivor  Participant #10 |
|  |  |  |
|  | 3c) Avoid seemingly arbitrary and senseless restrictions; they feel cruel and hurtful | *“They set a timer for us. One at a time, for fifteen minutes. They would not let us grieve together or be a family, they singled us out. And there was lots of room and no one was doing anything with, like she was stable then and comatose. There was no reason to treat us like that. It was terrible.”*  Daughter of survivor  Participant #14 |
|  |  |  |
|  | 3d) Remember to always attempt to contact and update families | *“Please call me to tell me this has happened. Like, please make some effort to let families know. I was at home, just doing whatever, not important and he is living his last moments and I don’t get to be a part of them because no one thinks to even call me. I have so much guilt because I was going about my life while my [partner] was having their last moments, alone, probably in pain, probably terrified and I was probably folding laundry in front of the television.”*  Partner of non-survivor  Participant #3 |
|  |  |  |
|  |  |  |
|  |  |  |
|  | 3e) Acknowledge us and our experience | *“I don't think any of them (health care workers) even talked to me about it. Nobody even said, “How are you?” or “How can we take care of you?” It also felt really strange like, it felt like there should have been something there but wasn't. It is the single worst experience of my life, I am forever changed and traumatised, and I’m in this hospital which is supposed to be a place of healing and there’s nothing for me? No help for me? I mean, come on. His heart is still beating, but we don’t know if he’s coming back from this and the best they can do is give me a chair and ignore me.”*  Son of survivor  Participant #13 |
|  | 3f) Acknowledge my loved one’s humanity through action | *“Before they left, they lifted him up off the floor and put him on the bed. And that felt really nice. They picked up all the garbage too and the paramedic supervisor wiped the blood from around his nose and mouth. That was so kind and thoughtful, right? They treated him like a person, not just some corpse. That felt so kind.”*  Partner of non-survivor  Participant #2 |
|  |  |  |
|  | 3g) Acknowledge my loved one’s humanity through words | *“And the ICU nurse, his name was X. He would talk with him about those pictures. And he was doing it in his nursing capacity. What he was doing was trying to find out what his cognitive ability and all of that was, right? But he did it in such a humane, compassionate way, as well, where he would just talk to him and just say, “Oh, are these your kids? What are their names? How old are they? And what school do they go to? Just things that are really designed to jog his memory and evaluate where his memories are, but also just talk to the patient about their life. And, you know, he was the only person I think that did that. And you know, without the family able to be there with him, that was it you know? And that has stuck with me as a really shining example of, of what to do and how to do it in those situations.”*  Partner of survivor  Participant #11 |
|  |  |  |
|  | 3h) Help us fulfil basic needs while we attend to our loved one | *“You know, like there, there weren't any chairs, and the lights weren't even on. It's just sort of like, it's almost like when you're in a classroom after school hours, and everything is put away. Yeah. It doesn't feel like a space that you're supposed to be in. It doesn’t feel like it is a space that’s supposed to be used.”*  Son of survivor  Participant #13 |
|  | 3i) Give us space to be a family together | *“I think the family room was actually nice. We charge our phones, we could have a cup of tea and be together. Having a place where we felt comfortable when we weren’t in the actual room, was really nice. And for the first night, they got me a recliner chair and a pillow and flannel and that was really great.”*  Daughter of survivor  Participant #16 |
|  |  |  |
|  | 3j) Provide us with simple and honest information | *“I think, I actually think you just need the truth. Yeah. I think you need the truth in a kind way. And I don't think it needs to be complicated. And I don't think you need it in a lot of words. And so I think that, um, you know, I think everybody's different, you know, but I actually think that confirming that worst scenario, in a simple way, kind way, was just exactly what we needed.”*  Mother of non-survivor  Participant #18 |
|  |  |  |
| 4) Address our family’s ongoing emergency | 4a) Help us manage our support network | *“One of the most important things, and difficult things, was for me to tell her parents and my parents and our work and our friends and to arrange for someone to get our kids. I needed to engage and manage our support systems. It was impossible and exhausting. My phone was essential and it was impossible to keep it charged. I remember begging for a charger for hours.”*  Partner of survivor  Participant #6 |
|  |  |  |
|  | 4b) Help us care for kids and dependents | *“I’ve got kids and my mother to take care of. And now my husband, he barely survived a cardiac arrest. Right? I needed a lot of help and I had none. Our house was a huge mess from the first responders, they had to break the door down. My kids were at school, my mother was at home alone, who knows what she understood. They all depend on me and I cannot be everywhere and be everything to everyone.”*  Partner of survivor  Participant #19 |
|  |  |  |
|  | 4c) Diffuse our acute grief and distress with us | *“So after they called the code they brought us into the room. All the doctors and nurses were walking out except for one. She said we could have twenty minutes to say goodbye and then we had to leave. I couldn’t believe it, I was so distraught, like we just had a total whirlwind. This was the worst day of our lives. I went from asleep to trying to resuscitate [female partner], to them dying in the hospital and we were being totally abandoned. We had no idea why she died, I couldn’t make sense of anything, I didn’t know what to do or what to think. I was wondering where they were going, because they’re not done, done. We still needed their help, but I don’t think they see it that way.”*  Partner of non-survivor  Participant #22 |
|  |  |  |
|  | 4d) Know that as our loved one’s needs lessen, ours may increase | *“You know, I’ll explain it. At first we needed help for [partner]. We needed the first responders and we needed all the hospital staff. We needed them to fix his heart. But we also needed help. After the initial cardiac arrest, our family was actually in crisis and as he got better, we actually got worse. And I don’t want to seem ungrateful because we are so fortunate, but after they saved [male partner], we needed someone to help save us.”*  Partner of survivor  Participant #21 |
|  |  |  |
| 5) Help us to heal, after the cardiac arrest | 5a) Navigate the after-arrest world | *“No, it has not ended. I think that it is a process that will never be done because, you know, we live with the consequences of our actions prior and post and so it's something that you live with the regret of the night before he left, you live with the regret of not going directly to the school and perhaps, you know, helping them apply the AED while he was still on the gym floor. And then afterwards it's years of dysfunction and broken family events because of missing him and, you know, grieving differently and fighting and divorce and so I think that it goes on way beyond what anybody realises and it impacts people's lives forever.”*  Mother of non-survivor  Participant #18 |
|  |  |  |
|  | 5b) Know it might get much worse before it gets better, even though things may look normal*  *note: 2 exemplary quotes chosen for their importance and poignancy | *“Yeah, and because the outcome was like [partner] literally walked out of the hospital 10 days later, people just kept saying how amazing his outcome was. But, I had to take the kids to therapy and get this therapist who did EMDR to help them with their, you know, healing process. I had to go to therapy. I had to get on anti anxiety medication because I wasn't doing well so yeah, it was definitely worse for me in that way. [partner] will say that over and over again, like it was more traumatic for me than him.”*  Partner of survivor  Participant #21 |
|  |  |  |
|  |  | *“We all went home after they moved him to the morgue and we had to leave the hospital. And we didn’t know what to do. We just faced the fact that a huge part of us, our rock, was never coming home again. [son] left the house, he went like AWOL for a week. [daughter] and I just sat together and cried for like a week. We just used the credit cards, we missed work, as we ordered food and cried and cried. It was the worst experience of our lives. Nothing compares.”*  Partner of non-survivor  Participant #5 |
|  | 5c) Learn how to be a caregiver | *“Boom! You’re responsible for so much. All of a sudden you’re a nurse and a parent, and a spouse, and a taxi driver and an accountant for insurance. It would have been great if someone could help to even make the follow-up appointments and even suggest a therapist or just offer that practical help. Because making those appointments is a more complicated cognitive task than what you have capacity for. I think, just putting this system into the standard way that things happen whenever there's a cardiac arrest is what is needed. I've spoken with a lot of doctors recently about all of this, and a lot of cardiologists and resuscitation doctors say, “Well, at my hospital, we have a chaplain or we have somebody who, who goes in and talks to you, like a social worker or whoever it is, and we really go above and beyond for our families and stuff.” And I'm like, “That’s great, but that's my point exactly. It should not be above and beyond that; it should just be the standard of care.”*  Partner of survivor  Participant #11 |
|  |  |  |
|  | 5d) Find our way in a disjointed healthcare system | *“We needed cardiology follow-up and neurological rehabilitation and job retraining. And we needed genetic testing and counselling. And we don’t even have a family doctor. So, we are at home and we don’t even know where to start. It is like, you’re discharged and then you are on your own to figure out what to do and I don’t even know what’s needed and what’s available.”*  Partner of survivor  Participant #15 |
|  | 5e) Arrange follow-up and aftercare | *“I kept thinking to myself, there should be a clinic for people like us. There should be more resources. Why should we have to do it all on our own? Spouses of survivors want to know, what's it like? What can you do? What should you do when you get home? What does it mean to be a caregiver? You know, I feel like I'm responsible for her entire, or his entire, life. Yes, like, if I mess up, they're gonna die. And without help, you have to be the advocate. Because you will fall through the cracks, and your surviving spouse will also.”*  Partner of survivor  Participant #20 |
|  |  |  |
|  | 5f) Be in the service of others | *“One of my sister’s friends, her husband had a cardiac arrest in the middle of the night at age 50, and survived. They live in [another province]. And she connected us because this woman was completely lost. We don't talk often, but every time we talk, I always feel better. Because I mean, there's some revisiting trauma, of course, but at the end of it, after it's processed, I feel empowered that I helped someone, and her response is always, nobody else makes me feel better, like you do.”*  Partner of survivor  Participant #12 |
|  |  |  |
|  | 5g) Find peer support and community | *“Peer support is the best, nothing could be more valuable. But, I do wonder if peer follow-up calls need to come from someone who's been there, and quite matched. So if your 90 year old has died you don't call someone whose 30 year old husband died, because it's just different, it just is. It's like a two year old versus an eighty year old dying. It's not the same thing. And for very different reasons. It's not the same thing. And you don't compare traumas; one is not worse than the other. It's just about relatability. But for myself, there's some empowerment and healing in being able to be there for someone else to say, this terrible thing happened to us. And I've turned it into a tool for good in some way. Right?”*  Partner of survivor  Participant #12 |
|  |  |  |
|  | 5h) Express gratitude for the care providers | *“It was very emotionally difficult for me. But those guys, I don't think get that kind of feedback very often. And I don't know what percentage of resuscitation they do actually survive, but it can’t be very many. And I've thanked all the physicians involved, and nursing staff, et cetera, and the ICU staff and, and all those kinds of folks. So that was actually a very important piece of the healing process. And something I would actually recommend for people if they didn't.”*  Survivor of  Participant #8 |
|  |  |  |
|  |  |  |

| **Table 7. Co-designed strategies for meeting the care needs of families experiencing cardiac arrest** | |
| --- | --- |
| Primary care need theme | Strategies |
| Help us help our loved one | Develop age-appropriate cardiac arrest awareness curricula for schools, from elementary to university level. Create engaging social media campaigns to raise awareness about cardiac arrest signs and symptoms. Organize regular community-wide CPR training events in partnership with local organizations. Implement AI-assisted dispatch systems to provide real-time, adaptive guidance for CPR. Develop a mobile app with offline capabilities for step-by-step CPR instructions and GPS functionality to alert nearby trained responders. Create virtual reality simulations for family members to practice CPR and using AEDs. Integrate cardiac arrest risk assessments into routine check-ups for all patients. Develop personalized emergency action plans for high-risk patients and their families. Create a "Cardiac Arrest Ready Home" certification program with home assessment checklists and in-home training. Advocate for legislation mandating CPR and AED training in schools and workplaces. Push for tax incentives for businesses that install AEDs and train staff. Launch a public campaign for placing AEDs in all public spaces, with clear signage and accessibility. Develop culturally sensitive, multilingual educational materials on activating emergency services. Create a program for families to practice "cardiac arrest drills" at home. Implement a follow-up program for families post-cardiac arrest event, offering debriefing and advanced training. Develop partnerships with workplaces to offer CPR and AED training as part of employee wellness programs. Create a national database of AED locations accessible via smartphone apps. Implement a system for automatic alerts to family members of high-risk individuals when a cardiac arrest is reported nearby. Develop a "CPR Coach" program where experienced individuals mentor others in their community. Create a recognition program for bystanders who perform CPR, highlighting their stories to inspire others. |
| Work with us, as a cohesive team | Develop protocols for assigning specific roles to capable family members during resuscitation efforts. Create a specialized training program for "Family Liaison Officers" in emergency and cardiac care units. Implement a real-time digital system for families to input information and concerns during ongoing care. Develop decision aids specifically designed for cardiac arrest scenarios to facilitate shared decision-making. Create a rapid ethics consultation service available 24/7 for complex cardiac arrest cases. Implement regular "Cardiac Arrest Care Improvement" forums involving healthcare providers, survivors, and families. Develop an online platform for continuous feedback and idea sharing between families and care teams. Create simulation scenarios focusing on difficult conversations and family interactions during cardiac crises. Implement a peer review system for provider-family communications during and after cardiac arrest events. Develop a rapid assessment tool to determine family readiness for presence during resuscitation. Create a "family support team" to assist those who choose to be present during resuscitation efforts. Implement a post-resuscitation debriefing protocol for families who were present during the event. Develop a comprehensive training curriculum for "Family Navigators" to guide families through the entire care journey. Create a standardized "Family Needs and Preferences" section in patient charts, regularly updated by the family. Implement a family-inclusive handover process at shift changes, allowing families to add notes or concerns. Develop a "Family Resuscitation Partner" training program for family members interested in being actively involved. Create regular "Cardiac Arrest Simulation Days" open to families to participate and provide feedback. Implement a system for families to access and contribute to the patient's medical record in real-time. Develop guidelines for automatic family conferences at key points in the cardiac arrest care journey. Create a "Family Advisor" role on hospital cardiac arrest committees to ensure the family perspective is always represented. |
| See us, treat us with humanity and dignity | Implement a structured format for family participation in daily care rounds, including a family preparation guide. Create private consultation rooms near cardiac care units designed with family comfort in mind. Implement a "comfort cart" service providing essential items to families during their hospital stay. Create multi-faith reflection spaces for spiritual and emotional support near cardiac care units. Develop a certification program in "Cardiac Crisis Family Support" for all staff in cardiac care areas. Implement regular emotional intelligence and trauma-informed care workshops for all healthcare staff. Create a standardized script and gesture for a "moment of pause" before and after resuscitation efforts. Develop a digital "family needs assessment" tool that can be updated in real-time by families and staff. Implement regular "family care conferences" to review and update the family needs assessment. Create a training curriculum for "Family Ambassadors" - experienced family members who support others. Develop a user-friendly app for real-time feedback from families on their care experience. Implement "feedback rounds" where staff actively seek family input on care and support. Create customizable "dignity kits" for families, including essential items and information resources. Develop a standardized protocol for breaking bad news in cardiac arrest situations, including follow-up support. Create an easily accessible digital platform for storing and updating end-of-life preferences. Implement a system to ensure all team members are aware of and respect patient and family preferences. Develop guidelines for staff on how to support families in performing cultural or religious practices. Create a "Family Support" team available 24/7 to address non-medical needs of families. Implement a program for staff to share positive stories and experiences of family-centered care. Develop a system for families to easily access clear, understandable explanations of all procedures and equipment. |
| Address our family’s ongoing emergency | Establish a 24/7 support hotline staffed by professionals trained in cardiac arrest crisis support. Develop a comprehensive "Family Care Package" with resources for managing practical, emotional, and legal aspects post-cardiac arrest. Create partnerships with community organizations to provide immediate support services (e.g., childcare, elder care, pet care). Implement a proactive outreach program with scheduled check-ins at key intervals post-event. Develop a risk assessment tool to identify families needing more intensive follow-up and support. Create a peer support network connecting families who have experienced cardiac arrest. Develop age-appropriate educational materials on managing stress, grief, and trauma for all family members. Establish a financial counselling service to help families navigate the economic impact of cardiac arrest. Develop a workplace education program to help employers support employees who are caregivers for cardiac arrest survivors. Create a mobile app for families to coordinate care tasks and access support resources. Implement a "family resilience" program teaching coping skills and stress management techniques. Develop a system for emergency responders to activate family support services immediately upon responding to a cardiac arrest. Create a "cardiac arrest family navigator" role to help families coordinate various aspects of care and support. Establish support groups specific to different family roles (e.g., spouses, children, parents). Develop a program to provide temporary housing for out-of-town families during prolonged hospital stays. Create a system for families to easily access and understand medical records and care plans. Implement a "family advocate" program to help families navigate healthcare and insurance systems. Develop partnerships with mental health providers for priority access to counselling services for affected families. Create a program to help families manage public and media attention in high-profile cases. Establish a grant program to help families cover non-medical expenses related to cardiac arrest care. |
| Help us to heal, after the cardiac arrest | Establish a long-term follow-up clinic for cardiac arrest survivors and their families, addressing physical, psychological, and social needs. Develop a structured rehabilitation program that includes family members in the recovery process. Create support groups specific to different family roles and outcomes (survivors, bereaved). Implement a comprehensive "life after cardiac arrest" education series covering adjusting to new roles, managing medications, and lifestyle changes. Develop resources and counselling services to address relationship challenges post-cardiac arrest. Create a mentorship program pairing experienced families with those new to the cardiac arrest journey. Establish an annual remembrance event for families to honour their loved ones and connect with others. Develop guidelines for primary care providers on long-term follow-up care for families affected by cardiac arrest. Create a research program that involves families in studying the long-term impacts of cardiac arrest on family dynamics and quality of life. Establish a grant program to support innovative community-based healing initiatives proposed by survivors and families. Develop a "cardiac arrest survivor and family" certification program for healthcare providers. Create a national database of cardiac arrest survivors and families for ongoing research and support. Implement a program for cardiac arrest survivors and families to speak at medical conferences and training events. Develop a "life transition" support program for survivors returning to work or school. Create a public awareness campaign to educate communities about the long-term effects of cardiac arrest on survivors and families. Establish a legal support service to help families navigate disability claims and insurance issues. Develop a program to support siblings of young cardiac arrest survivors or victims. Create a network of "cardiac arrest friendly" employers who understand the unique needs of survivors and family caregivers. Implement a system for long-term monitoring of psychological health in survivors and family members. Develop a program to support families in advocating for improved cardiac arrest care and prevention policies. |

**Interview Questions**

Hello!

Thank you for agreeing to participate in this interview. Before we get started, do you have any questions?

I want to remind you that you can stop the interview at any time and if you do not want to answer a question, that’s perfectly ok.

If you find the interview emotionally distressing, we can stop the interview and there are mental health professionals available for you to access.

If you are ready, we will begin.

Who in your family experienced cardiac arrest, what is your relationship to them?

Is there anything you would like to tell me about them?

Did they survive the arrest?

When would you say the event, the cardiac arrest started and when was it over?

Based on your experience, what do family members need during the cardiac arrest care of a loved one?

Physical items?

Intangible things?

Were there different phases of care that families require different care during?

Prehospital, ED, ICU?

Prehospital, ED, morgue?

Prehospital, morgue?

Were you present during the cardiac arrest?

Did you provide care to your loved one?

Were you present during the cardiac arrest care by paramedics, emergency department or intensive care staff?

What do you think health care professionals such as paramedics, emergency department staff, intensive care staff, social workers and pastoral care should provide (or do for) families during the cardiac arrest of a loved one?

Prehospital, ED, ICU?

Prehospital, ED, morgue?

Prehospital, morgue?

More and more often people’s last moments are captured on video. Would you have liked to have video of your loved one’s cardiac arrest and care shared with you? How would you feel about researchers and health care workers using these video to try and improve cardiac arrest care and help families with their grief?

When an infant dies in the hospital, families are often provide mementos like hand and foot imprints. Would such things be of value to you if health care workers provided them for you?

Do you think there’s anything else I should know, related to these questions? Is there anything you would like to add to a previous answer? Would you like to review the questions at all?

That concludes this interview.

I want to remind you that you may withdrawal your interview from the study up until the start of data analysis. To withdrawal, all you need to do is call or email me. Do you need my contact info again?

Thank you for your time,

MJD

| Table 8. Patient and Public Involvement in the Supporting Family-Centred Cardiac Arrest Care Project  GRIPP2 Short Form. | |
| --- | --- |
| Aim | To perform an interview study and qualitative analyses with cardiac arrest survivor and family members as co-investigators. The study aimed to describe the care needs of families experiencing cardiac arrest and identify strategies to meet those care needs. |
| Methods | Survivor and family partners were recruited from an existing Family Centred Cardiac Arrest Care online working group and through social media. These partners were involved in multiple stages of the research process:  Conception of the project  Refining the focus of the research questions  Recruiting participants  Co-coding transcripts  Developing the codebook  Co-analyzing the data  Manuscript preparation  Dissemination of the project findings |
| Results | Survivor and family co-investigator and collaborator involvement in this project was effective and resulted in many substantial contributions. Their involvement from the project's inception allowed them to help shape the methods, analysis, and findings of the work.  Pre-existing relationships with potential survivor and family co-investigators proved to be an efficient and effective method for recruitment.  Limitations included:  Lack of an online portal or forum for improved asynchronous work  Absence of monetary honorariums for co-researchers  Time-intensive nature of co-analysis |
| Discussion | Survivor and family co-investigator and collaborator involvement in this project was effective and they made many substantial contributions. The survivor and family co-investigators were involved from the beginning of the project allowing them to help shape the methods, analysis and findings of this work. Pre-existing relationships with potential survivor and family co-investigators were an efficient and effective method for their recruitment to collaborate. However, there were limitations, having an online portal or forum could improve asynchronous work as the volume and frequency of emails was deemed to be too great for the PI to keep up with at times. Furthermore, monetary honorariums for co-researchers would have helped value their contributions, especially since co-analysis is incredibly time consuming. |
| Reflections | The survivor and family co-investigators and collaborators were engaged as co-researchers, able to co-lead the project. This level of involvement was a strength of the study.  The project was unfunded, and additional research support and coordination (including administrative and technology support) would have aided in its execution.  There was a large amount of work done by a small group and a small amount of work done by a larger group who engaged more transiently. The contributions of both groups were valuable to the process.  Future similar projects could benefit from:   - Implementing an online collaboration platform - Securing additional funding for honorariums and administrative support - Carefully planning for the time-intensive nature of co-analysis |
| GRIPP2  Source | http://wrap.warwick.ac.uk/91556/1/WRAP-GRIPP2-reporting-checklists-Staniszewska-2017.pdf |

|  | Table 9. Standards for Reporting Qualitative Research (SRQR)* |
| --- | --- |
|  | S1 Title: Concise description of the nature and topic of the study. Identifying the study as qualitative or indicating the approach (e.g., ethnography, grounded theory) or data collection methods (e.g., interview, focus group) is recommended. (Page 1)  S2 Abstract: Summary of key elements of the study using the abstract format of the intended publication; typically includes background, purpose, methods, results, and conclusions. (Page 1)  S3 Problem formulation: Description and significance of the problem/phenomenon studied; review of relevant theory and empirical work; problem statement. (Pages 2-3)  S4 Purpose or research question: Purpose of the study and specific objectives or questions. (Page 3)  S5 Qualitative approach and research paradigm: Qualitative approach (e.g., ethnography, grounded theory, case study, phenomenology, narrative research) and guiding theory if appropriate; identifying the research paradigm (e.g., postpositivist, constructivist/ interpretivist) is also recommended; rationale. (Pages 3-4)  S6 Researcher characteristics and reflexivity: Researchers' characteristics that may influence the research, including personal attributes, qualifications/experience, relationship with participants, assumptions, and/or presuppositions; potential or actual interaction between researchers' characteristics and the research questions, approach, methods, results, and/or transferability. (Page 4)  S7 Context: Setting/site and salient contextual factors; rationale. (Page 4)  S8 Sampling strategy: How and why research participants, documents, or events were selected; criteria for deciding when no further sampling was necessary (e.g., sampling saturation); rationale. (Page 4)  S9 Ethical issues pertaining to human subjects: Documentation of approval by an appropriate ethics review board and participant consent, or explanation for lack thereof; other confidentiality and data security issues. (Page 6)  S10 Data collection methods: Types of data collected; details of data collection procedures including (as appropriate) start and stop dates of data collection and analysis, iterative process, triangulation of sources/methods, and modification of procedures in response to evolving study findings; rationale. (Pages 4-5)  S11 Data collection instruments and technologies: Description of instruments (e.g., interview guides, questionnaires) and devices (e.g., audio recorders) used for data collection; if/how the instrument(s) changed over the course of the study. (Pages 4-5)  S12 Units of study: Number and relevant characteristics of participants, documents, or events included in the study; level of participation (could be reported in results). (Pages 6-7)  S13 Data processing: Methods for processing data prior to and during analysis, including transcription, data entry, data management and security, verification of data integrity, data coding, and anonymization/de-identification of excerpts. (Page 5)  S14 Data analysis: Process by which inferences, themes, etc., were identified and developed, including the researchers involved in data analysis; usually references a specific paradigm or approach; rationale. (Pages 5-6)  S15 Techniques to enhance trustworthiness: Techniques to enhance trustworthiness and credibility of data analysis (e.g., member checking, audit trail, triangulation); rationale. (Pages 5-6)  S16 Synthesis and interpretation: Main findings (e.g., interpretations, inferences, and themes); might include development of a theory or model, or integration with prior research or theory. (Pages 7-10)  S17 Links to empirical data: Evidence (e.g., quotes, field notes, text excerpts, photographs) to substantiate analytic findings. (Pages 7-10)  S18 Integration with prior work, implications, transferability, and contribution(s) to the field: Short summary of main findings; explanation of how findings and conclusions connect to, support, elaborate on, or challenge conclusions of earlier scholarship; discussion of scope of application/generalizability; identification of unique contribution(s) to scholarship in a discipline or field. (Pages 10-12)  S19 Limitations: Trustworthiness and limitations of findings. (Page 13)  S20 Conflicts of interest: Potential sources of influence or perceived influence on study conduct and conclusions; how these were managed. (Page 13)  S21 Funding: Sources of funding and other support; role of funders in data collection, interpretation, and reporting. (Page 13) |
|  | **Reference:** |
|  | O'Brien BC, Harris IB, Beckman TJ, Reed DA, Cook DA. **Standards for reporting qualitative research: a synthesis of recommendations.** *Academic Medicine*, Vol. 89, No. 9 / Sept 2014  DOI: 10.1097/ACM.0000000000000388 |
|  |  |
|  |  |
